# Herpes Simplex Virus Hepatitis in an Immunocompetent Adult: A Fatal Outcome due to Liver Failure

**DOI:** 10.1155/2011/138341

**Published:** 2011-12-07

**Authors:** Rachel A. Poley, Jaime F. Snowdon, Daniel W. Howes

**Affiliations:** ^1^Department of Emergency Medicine and Department of Critical Care Medicine, Kingston General Hospital, Queen's University, Kingston, ON, Canada K7L 2V7; ^2^Department of Pathology, Queen's University, Kingston, ON, Canada K7L 3N6

## Abstract

*Objective*. To present a case of a healthy 41-year-old female who developed fulminant hepatic failure leading to death. The cause of hepatic failure identified on postmortem exam was herpes simplex virus hepatitis. *Design*. Observation of a single patient. *Setting*. Intensive care unit of a tertiary care university teaching hospital in Canada. *Patient*. 41-year-old previously healthy female presenting with a nonspecific viral illness and systemic inflammatory response syndrome. *Intervention*. The patient was treated with intravenous fluids and broad-spectrum antibiotics. On the second day of admission, she was found to have elevated transaminases, and, over 48 hours, she progressed to fulminant liver failure with disseminated intravascular coagulopathy, refractory lactic acidosis, and shock. She progressed to respiratory failure requiring intubation and mechanical ventilation. She was started on N-acetylcysteine, a bicarbonate infusion, hemodialysis, and multiple vasopressors and inotropes. *Measurements and Main Results*. Despite treatment, the patient died roughly 70 hours after her initial presentation to hospital. Her postmortem liver biopsy revealed herpes simplex virus hepatitis as her cause of death. *Conclusions*. Herpes simplex virus must be considered in all patients presenting with liver failure of unknown cause. If suspected, prompt treatment with acyclovir should be initiated.

## 1. Introduction

We present the case of a previously healthy 41-year-old female who developed herpes simplex virus hepatitis leading to fulminant liver failure and death. Antemortem diagnosis of this disease is often difficult, and, as a result proper treatment is delayed. The aim of this paper is to review this rare but important cause of liver failure, its clinical presentation, and available treatment options.

## 2. The Case

A 41-year-old female presented ambulatory to the Emergency Department at 11:41 PM with a 3-week history of feeling generally unwell. She complained of severe myalgias, chills, and a fever two days earlier. She had been unable to go to work for the previous 4 days due to her illness. She admitted to taking ibuprofen, up to 800 mg every 2 hours for several days for her myalgias, but she and her husband denied any significant acetaminophen ingestion. She was hemodynamically unstable on presentation with a heart rate of 114 beats per minute and a blood pressure of 71/40 mm Hg. She was afebrile with a temperature of 36.4°C. Her respiratory rate was 18 breaths per minute, and her oxygen saturations were 98% on room air. She was alert and oriented with a GCS of 15 and was able to give a history of her illness. Her head and neck exam was unremarkable, and no oral lesions were documented. Her respiratory and cardiac examinations were normal. Her abdomen was soft and nontender with no organomegaly, and her skin was clear. A pelvic exam was not performed. Her past medical history was significant only for two uncomplicated pregnancies, a tubal ligation, and dysfunctional uterine bleeding. Her only medication was an oral contraceptive pill. Her social history was significant for 26 ounces of alcohol per week, mostly on weekends. There was no history of smoking, drug use, travel, or high-risk sexual behaviors.

Her investigations were significant for acute renal failure with a creatinine of 214 umol/L. Her white blood cell count was 7.2 × 10^9^/L, hemoglobin was 130 g/L, and platelets were slightly decreased at 115 × 10^9^/L. Her lactate was 1.5 mmol/L, and her venous blood gas showed a mild compensated metabolic acidosis with a ph of 7.32, a CO2 of 39 mm Hg, and a HCO3 of 20 mmol/L. Her urine b-HCG was negative. Liver function studies and liver enzymes were not performed on initial presentation.

No focus for infection was found, and she was presumed to have a viral illness with a systemic inflammatory response syndrome (SIRS). She was treated with intravenous fluid administration, and she was given broad-spectrum antibiotics to cover for any bacterial pathogen that may be causing her SIRS. The next morning a liver panel was ordered and showed significantly elevated transaminases (AST 4090 U/L, ALT 1692 U/L, ALP 123 U/L, total bilirubin 24 umol/L, INR 2.3, PT 25.9 seconds, PTT 91 seconds, platelets 85 × 10^9^/L) with signs of liver failure and disseminated intravascular coagulopathy but a relatively normal bilirubin. She was now leukopenic with a white blood cell count of 3.6 × 10^9^/L. Her acetaminophen level later that day was undetectable. Regardless, she was started on an N-acetylcysteine infusion as treatment for a possible late presentation of acetaminophen toxicity. Her ultrasound showed gallbladder wall thickening (5 mm) but a negative sonographic Murphy's signs; the portal vein was patent. The imaging was considered consistent with hepatitis. She was initially thought to have liver dysfunction due to prolonged hypotension on presentation resulting in hepatic ischemia. Throughout that day, the patient developed fulminant liver failure with a severe lactic acidosis and disseminated intravascular coagulopathy and required intubation. She was seen by multiple specialists throughout the course of the day and was considered for liver transplant but was deemed unsuitable. No cause for her liver failure was identified. She died later that day after developing vasopressor refractory hypotension, roughly 70 hours after presentation.

**Figure 1:**
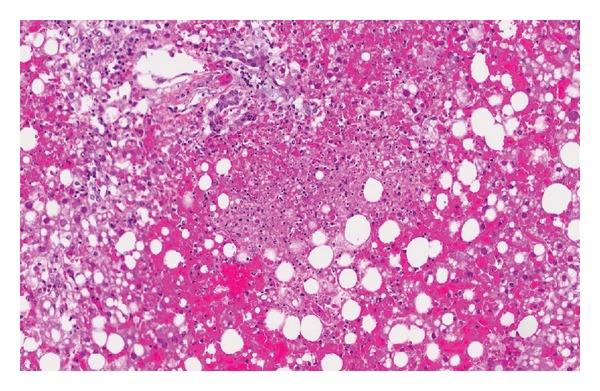
Zones of hepatocyte necrosis surrounded by hemorrhage without significant inflammation. (100x magnification; Hematoxylin-Phloxine-Saffron (HPS) stain.)

**Figure 2:**
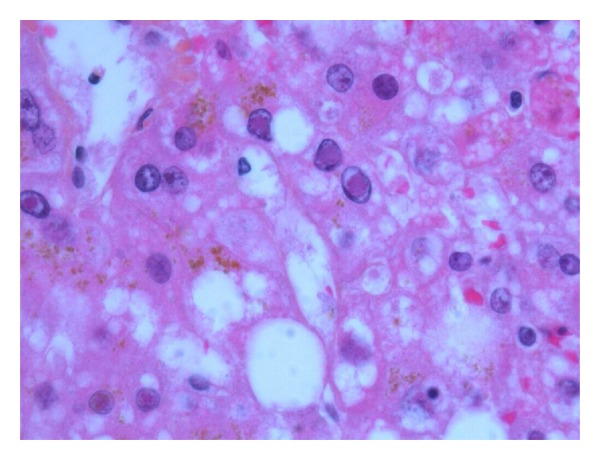
Viral inclusions are readily visible in infected hepatocytes. (600x magnification; Hematoxylin-Phloxine-Saffron (HPS) stain.)

**Figure 3:**
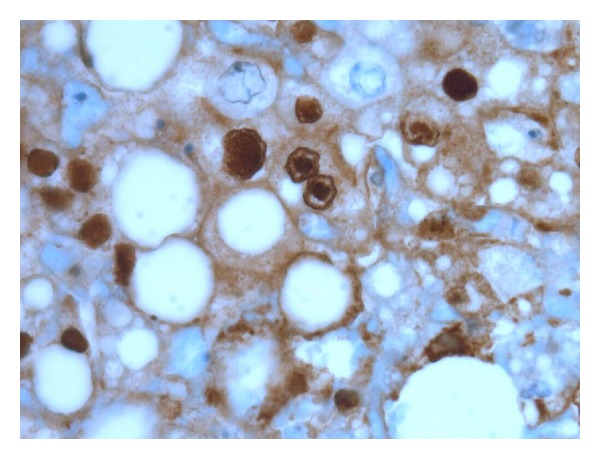
Immunohistochemistry for HSV virus highlights viral inclusions (600x magnification).

Serologic test results were available shortly after her death. She was negative for hepatitis A, B, and C viruses (hepatitis B surface antigen nonreactive and surface antibody reactive, hepatitis A IgG antibody nonreactive and IgM antibody nonreactive, hepatitis C antibody nonreactive). She was screened for multiple additional viruses including parvovirus b19, cytomegalovirus, Ebstein-Barr virus, and all of these were negative. On her postmortem liver biopsy, she had signs of severe acute liver necrosis. There was no evidence of chronic underlying liver disease. All other organ systems were spared. Tissue samples from her liver were sent for multiple tests and were negative for influenza, adenovirus, enterovirus, rhinovirus, parainfluenza virus, metapneumovirus, corona virus, and respiratory syncytial virus. Her polymerase chain reaction (PCR) testing was negative for cytomegalovirus virus and varicella zoster but positive for herpes simplex virus (HSV) type 1. Viral culture of her liver sample was positive for HSV type 1. Liver histology revealed diffuse acute liver necrosis with few inflammatory changes (Figure 1). Multiple viral inclusion bodies with the characteristic clear halo distinctive of Cowdry type A inclusions were seen in her hepatocytes (Figure 2). Immunostaining was positive for HSV antibodies (Figure 3). There was no evidence of disseminated HSV. Her cause of death was determined to be herpes simplex viral hepatitis causing fulminant liver failure.

Our Institutional Research Ethics Board has approved this case report, and the need for informed consent has been waived.

## 3. Discussion

Herpes simplex virus (HSV) is extremely common throughout North America and the world. It is estimated that up to 80% of adults contract HSV throughout their lifetime [1] and that most infections are asymptomatic or produce only mild nonspecific viral symptoms. HSV hepatitis is rare and accounts for only 1% of all acute liver failure cases and only 2% of all viral causes of acute liver failure (ALF) [1, 2]. It occurs most commonly in organ transplant patients, in the third trimester of pregnancy or in patients who are otherwise immunocompromised, but up to 25% of patients who develop HSV hepatitis are immunocompetent [3].

HSV hepatitis presents with nonspecific flu-like symptoms including fever, myalgias, and abdominal pain. Only 30–50% show characteristic herpetic skin lesions [3–7]. Laboratory investigations often show leucopenia, thrombocytopenia, and coagulopathy [3–6, 8–10]. Renal failure is not uncommon in these patients [5, 11–14], and it has been shown to occur in up to 65% of patients with HSV-related ALF [3]. Disseminated intravascular coagulopathy is frequently reported [5, 8, 9, 15, 16], and encephalopathy is a late sign of the disease. Ninety percent of patients with HSV hepatitis have a characteristic liver profile, known as “anicteric hepatitis” [6, 9, 17, 18]. Anicteric hepatitis refers to a liver profile showing a significant increase in transaminases (100–1000 fold) with a relatively normal or low bilirubin [1, 3, 6, 9, 16]. There may be a marked elevation of AST greater than ALT [6].

Antemortem diagnosis of HSV hepatitis is difficult and is considered in only 23–42% of cases prior to autopsy [3, 15, 19]. Investigations to aid in the diagnosis for HSV hepatitis are limited. Viral serology cultures are extremely sensitive and can be used as a screening tool but are very poorly specific. Viral PCR testing may be useful but is often not rapidly available. Although not always possible due to coagulopathy, the gold standard for diagnosis is liver biopsy. Cowdry type A inclusions, nuclei with large eosinophilic ground glass-like inclusions surrounded by a clear halo, are pathognomonic for HSV hepatitis [8, 15]. Histology shows extensive areas of hepatocyte necrosis with adjacent congestion but minimal inflammatory infiltrates [20]. Immunohistochemical staining can be done to confirm the diagnosis of HSV, and the presence of viral antigens can be demonstrated by immunoperoxidase staining and by identifying monoclonal antibodies against HSV antigens [20].

HSV hepatitis leads to ALF in 74% of cases, and, in these cases, the mortality rate reaches up to 90% [1, 3, 6, 19, 21]. Antiviral treatment with acyclovir has been used successfully [3, 5–7, 14, 17, 19, 22–25]. The extent of disease at the initiation of acyclovir plays a large role in its effectiveness, but outcomes probably improve with earlier initiation of therapy [3, 8, 15, 22]. Many authors recommend empiric treatment with acyclovir in patients with ALF of unknown origin [1, 3, 6, 8]. High urgency liver transplant should also be considered early in the course of the disease as it has shown to improve outcomes [3, 8, 22, 23].

Physicians should consider HSV hepatitis in patients with fulminant liver failure of unknown cause. A thorough examination, including examination of the oropharynx and a complete pelvic exam, may provide clues to the diagnosis. If possible, liver biopsy is the gold standard for diagnosis. In any patient presenting with flu-like illness and anicteric hepatitis, HSV should be suspected and early treatment with acyclovir should be strongly considered.
